# Clinically relevant characteristics associated with early treatment drug use versus abstinence

**DOI:** 10.1186/1940-0640-9-6

**Published:** 2014-04-04

**Authors:** Gerald Cochran, Maxine Stitzer, Edward V Nunes, Mei-Chen Hu, Aimee Campbell

**Affiliations:** 1 University of Pittsburgh, School of Social Work, 4200 Forbes Avenue, 2117 CL, 15260 Pittsburgh, PA, USA; 2 Department of Psychiatry and Behavioral Sciences, The Johns Hopkins University School of Medicine, Meyer 4-113, 600 North Wolfe Street, 21287-7413 Baltimore, MD, USA; 3 Department of Psychiatry, New York State Psychiatric Institute, 1051 Riverside Drive, 10032 New York, NY, USA; 4 Columbia University, College of Physicians and Surgeons, 630 West 168th Street, 10032 New York, NY, USA; 5 Department of Psychiatry and Behavioral Health, Mount Sinai St. Luke’s, 1111 Amsterdam Avenue, 10025 New York, NY, USA

**Keywords:** Substance abuse, Substance abuse treatment, Co-occurring disorders, Co-morbidity, Screening

## Abstract

**Background:**

This study describes early treatment drug use status and associated clinical characteristics in a diverse sample of patients entering outpatient substance abuse psychosocial counseling treatment. The goal is to more fully characterize those entering treatment with and without active use of their primary drug in order to better understand associated treatment needs and resilience factors.

**Methods:**

We examined baseline data from a NIDA Clinical Trials Network (CTN) study (Web-delivery of Treatment for Substance Use) with an all-comers sample of patients (N = 494) entering 10 outpatient treatment centers. Patients were categorized according to self-identified primary drug of abuse (alcohol, cocaine/stimulants, opioids, marijuana) and by baseline drug use status (positive/negative) based on urine testing or self-reports of recent use (alcohol). Characteristics were examined by primary drug and early use status.

**Results:**

Classified as drug-negative were 84%, 76%, 62%, and 33% of primary opioid, stimulant, alcohol, and marijuana users; respectively. Drug-positive versus -negative patients did not differ on demographics or rates of substance abuse/dependence diagnoses. However, those negative for active use had better physical and mental health profiles, were less likely to be using a secondary drug, and were more likely to be attending 12-step self-help meetings.

**Conclusions:**

Early treatment drug abstinence is common among substance users entering outpatient psychosocial counseling programs, regardless of primary abused drug. Abstinence (by negative UA) is associated with better health and mental health profiles, less secondary drug use, and more days of 12-step attendance. These data highlight differential treatment needs and resiliencies associated with early treatment drug use status.

**Trial registration:**

NCT01104805.

## Background

Outpatient substance abuse treatment programs that offer psychosocial counseling as the primary care modality enroll individuals who present for treatment with a wide range of drug use profiles. Prominent among these individual differences are the drug(s) endorsed as the primary problem, and reason for treatment entry, and the current drug use status of the individuals entering treatment. In clinical trials with stimulant abusers, it repeatedly has been shown that a substantial proportion has already achieved abstinence prior to treatment entry or does so rapidly upon treatment entry, and that early drug abstinence is strongly associated with better treatment prognosis [1-16]. Studies among alcohol users receiving treatment similarly have described divergent pre-treatment drinking patterns, including pre-treatment abstinence, and have demonstrated their association with treatment outcomes [17,18]. However, these studies infrequently report the physical, mental health, and behavioral characteristics of individuals entering treatment as a function of either current drug use status or primary drug problem. Given the major emphasis in health-care reform regarding the identification and treatment of co-occurring health, mental health, and substance use disorders [19,20], it is critical to expand current knowledge in the field regarding baseline health and behavioral health profiles of individuals entering substance abuse treatment in a more nuanced and detailed fashion.

A recently completed study within the National Institute on Drug Abuse Treatment Clinical Trials Network (NIDA CTN 0044; *WEB-TX*) enrolled a large “all-comers” sample of substance users seeking treatment at 10 regionally diverse outpatient psychosocial counseling programs for an array of specific primary substance use problems; including problems associated with alcohol, cocaine/stimulants, opioids, marijuana. This large and diverse sample provides the opportunity to obtain a comprehensive profile of pre- and early-treatment behavioral health, substance use, and behavioral resilience factors among a large and diverse sample of substance users and to relate these variables to drug use status (active-use versus abstinence) shortly after treatment entry. Such information has the potential to inform providers about patients’ needs and challenges when initiating treatment in order to better tailor care for those needs.

## Methods

### Participants and recruitment procedures

Methods of this CTN clinical trial have been described in detail elsewhere [21]. Here, we present a brief overview with emphasis on methods relevant to the current secondary analysis of baseline data. Individuals seeking substance abuse treatment at 10 regionally diverse outpatient treatment centers across the US were given information on the *WEB-TX* study at treatment entry and, if interested, were referred to research staff members at the treatment program. After providing verbal consent, potential participants were assessed for eligibility via a short screening instrument. If the participant was eligible, a baseline assessment was scheduled. Before completing the baseline assessment, patients provided written informed consent. The study was designed to maximize participant heterogeneity and recruit all-comers seeking substance abuse treatment; thus, eligibility was kept purposefully broad.

Eligible participants: 1) were 18 years of age or older; 2) had self-reported a substance use problem and illicit substance use in the 30 days prior to baseline assessment (or 60 days if the participant was exiting a controlled environment); 3) were randomized within the first 30 days of the outpatient treatment episode; and 4) had planned a treatment episode of 3 months or more. Patients were ineligible for the study if they: 1) were undergoing opioid treatment or were receiving opioid substitution pharmacotherapy; 2) were planning to move out of the area within 90 days; 3) were not able to provide informed consent; and 4) did not speak English. Of the 507 randomized to the study, 13 participants were excluded from the current analyses; specifically, 10 were excluded because their primary substance of abuse was not one of those analyzed here (i.e., nine benzodiazepine and one PCP), and three (two marijuana and one stimulant) were excluded because of missing baseline urine test data [22]. This study was approved by and conducted in accordance with the standards of the institutional review board of each participating treatment program. Review Boards included those from: New York State Psychiatric Institute, University of Miami, University of Washington, John Hopkins Medicine, Bio-Med IRB, UT Southwestern Medical Center, University of Cincinnati, Yale University, Long Island Jewish Medical Center, McLean Hospital, and Oregon Health & Science University.

### Baseline assessment procedures

Baseline assessments were conducted by trained research staff members at the treatment programs. The mean time between treatment entry and baseline assessment was 9 days (SD = 7.1 days), with no significant differences by primary drug class or drug use status at baseline. The baseline assessment took approximately 2 hours to complete.

### Measures

#### ***Demographic variables***

The demographic variables captured were gender, age, race/ethnicity, education, marital status, employment status, and whether the current treatment episode was mandated by the criminal-justice system.

#### ***Substance use***

Substance abuse and dependence were assessed using the DSM-IV Checklist, a semi-structured interview that provides current (past year) diagnosis for substance use disorders based on DSM-IV criteria [23]. Patients also were asked to report their primary substance of abuse (i.e., the substance of primary concern for which treatment was sought) and to report other substances used in the prior 12 months. Frequency of use of each substance mentioned was assessed over the 90 days prior to baseline using the Timeline Follow-back (TLFB) tool [24], and data were summarized for self-reported use in the past 7 and past 30 days.

#### ***12-step attendance***

Participants were asked whether or not they had attended any 12-step groups or meetings in the 90 days prior to baseline assessment and if so, how many days they had attended.

#### ***Physical and mental health***

Physical health was assessed using a visual analogue scale from the Euro Quality of Life Scale-EQ5D [25]. Scores on this instrument range from 0 to 100, with higher scores representing better health. Mental health was assessed using the Patient Health Questionnaire (PHQ), which results in probable diagnoses across six psychiatric disorders including: major depression, generalized anxiety, and panic disorders [26].

#### ***Psychosocial functioning***

Social functioning was assessed using the Social Adjustment Scale [27]. This 54-item instrument assesses social role functioning in the domains of work, social and leisure activities, family relationship, marital relationship, parental role, and role within the family unit. Items are rated on a 5-point scale, with lower numbers indicating higher functioning.

#### ***Statistical methods***

Participants (N = 494) were divided into four categories based on their self-reported primary drug of abuse: alcohol (n = 104), marijuana (n = 112), cocaine/stimulants (n = 170) and opioids (n = 108). Initial drug use status was based on results of study intake urine tests (positive versus negative) for the primary substance. Positive urine tests for secondary drugs were recorded but ignored for purposes of participant classification. In the case of those seeking treatment for alcohol, classification was based on self-report of any alcohol use (yes or no) during the 7 days prior to baseline, due to the very short detection window of breath alcohol testing that resulted in a low frequency of positive Breathalyzer results at baseline (n = 3) among primary alcohol users.

Chi-square tests for categorical variables and t-tests for continuous variables were employed to analyze the data as a function of drug use status at study entry and primary drug category using SAS 9.2 [28]. Pair-wise comparisons were conducted between positive versus negative participants within each primary substance subgroup.

## Results

### Demographic characteristics

Table 1 shows baseline demographic variables for the overall study sample (N = 494). Data is collapsed across primary drug use and baseline positive/negative categories because no differences were found on any demographic variable shown for drug-positive versus -negative participants within each primary drug category or within the drug-positive versus -negative samples overall. The one exception was for race in the overall sample, in which those testing positive (26.7% African American; 15.1% Hispanic) were more likely than those testing negative (19.6% African American; 8.7% Hispanic) to be from an ethnic minority group (*p* < .05 and; *p* < .01).

**Table 1:** Baseline participant characteristics (N = 494)

**Characteristic**	**%**
Female	38%
Age^a^	35 (11)
Race	52%
White	
African American	22%
Hispanic Latino	11%
Other	15%
Education	23%
Less than High School	
High School/GED	61%
More than High School	15%
Marital Status	60%
Single, Never Married	
Married/Remarried	14%
Divorced/Separated/Widowed	26%
Employed	41%
Insured	76%
CJ Tx Status	
No	65%
Mandated	21%
Referred or Recommend to Tx	14%

^a^Mean years and SD.

### Baseline positive/negative drug use status

Table 2 shows rates of participants abstinent by urine test results for their primary drug, as well as rates self-reporting any use of that drug in the previous 30 days. Rate of participants classified as abstinent by negative urine test at study start was 84% for primary opioid users, 76% for stimulant users, 62% for those claiming alcohol as their primary drug (by self-report of use in past 7 days), and 33% for primary marijuana users. Self-report of any use in the past 30 days was highly congruent with urine test results for those testing drug-positive. Among those testing drug-negative, however, 62-81% across the primary drug categories reported having used their primary drug within the past 30 days. Chi-square tests indicated that rates of self-reported use versus negative urine test results were significantly discrepant for all primary drug categories.

**Table 2:** Urine/breath baseline use status

		**Self-report drug use in previous 30 days**				
		**No**	**Yes**	**χ**^**2**^	**df**	***p***
**Drug**	**Urine drug-positive**	**% (n)**	**% (n)**			
Alcoho^a^	No	35.9 (23)	64.06 (41)	18.5	1	<.001
	Yes	0 (0)	100 (40)			
Marijuana	No	18.9 (7)	81.1 (30)	11.6	1	<.001
	Yes	1.3 (1)	98.7 (74)			
Cocaine/Stimulants	No	31.0 (40)	68.0 (89)	13.9	1	<.001
	Yes	2.4 (1)	97.6 (40)			
Opioids	No	37.4 (34)	62.2 (57)	9.3	1	<.001
	Yes	0 (0)	100 (17)			

^a^Biological validation of alcohol use based on Breathalyzer and on self-report of alcohol use in the past 7 days.

More detailed data about recent drug use can be derived from days of self-reported drug use in the past 30 days. Across all primary drug categories, those classified as positive at baseline for their primary drug reported more days of use of that primary drug in the past 30 days than did those classified as negative (results not shown). Days of use for those testing positive versus negative were 10.9 versus 5.5 for primary alcohol users (*t* = 3.70*; p* < .001), 18.1 versus 7.1 for primary marijuana users (*t* = 5.74; *p* < .001), 12.4 versus 3.6 for primary stimulant users (*t =* 5.80; *p* < .001), and 9.7 versus 4.8 for primary opioid users (*t =* 2.79; *p* < .01).

A different perspective on drug use status is provided by patterns of past year substance abuse or dependence, as shown in Table 3. Percent of participants meeting criteria for abuse or dependence on their primary drug was 95-100% across all primary drug categories except marijuana, where rates of abuse or dependence were slightly lower, at 80-90%. Importantly, rates of DSM abuse or dependence were not significantly different for those testing positive versus negative for their primary drug at study start.

**Table 3:** Substance abuse or dependence diagnosis and secondary use

	**Percent qualifying for diagnosis**											
	**Alcohol**			**Marijuana**			**Cocaine/Stimulants**			**Opioids**		
	**Neg N = 64**	**Pos N = 40**	**(χ**^**2 **^**[df], *p*)**	**Neg N = 37**	**Pos N = 75**	**(χ**^**2 **^**[df], *p*)**	**Neg N = 129**	**Pos N = 41**	**(χ**^**2 **^**[df], *p*)**	**Neg N = 91**	**Pos N = 17**	**(χ**^**2 **^**[df], *p*)**
Abuse/Dependence												
Alcohol	95.3	100.0	(1.93 [1], 0.16)	56.7	48.0	(0.76 [1], 0.38)	55.8	51.2	(1.93 [1], 0.16)	56.0	47.1	(1.93 [1], 0.16)
Marijuana	51.6	42.5	(0.81 [1], 0.37)	81.1	89.3	(1.45 [1], 0.23)	33.3	34.2	(0.01 [1], 0.92)	38.5	29.4	(0.50 [1], 0.48)
Cocaine/ Stimulants	51.6	45.0	(0.42 [1], 0.51)	21.6	24.0	(0.08 [1], 0.80)	98.5	95.1	(1.50 [1], 0.22)	45.1	35.3	(0.56 [1], 0.46)
Opioids	32.8	22.5	(1.28 [1], 0.26)	8.1	6.7	(0.08 [1], 0.78)	11.6	9.8	(0.11 [1], 0.74)	98.9	100.0	(0.19 [1], 0.66)
Non-primary substance use at baseline^a^	21.9	50.0	(8.85 [1], 0.003)	29.7	46.7	(2.94 [1], 0.09)	17.8	51.2	(18.08, <.0001)	26.4	23.5	(0.06 [1], 0.81)

^a^Alcohol use identified by self-report of any use in past 7 days; other drugs identified by positive urine test.

### Secondary drug use

Table 3 also shows that the percentage of participants meeting criteria for past year abuse or dependence on non-primary drugs, which was common. Consistent with study entry criteria, those with a primary alcohol problem qualified at substantial rates for abuse or dependence on other drugs (48% marijuana, 49% cocaine/stimulant, 29% opioid; collapsed across positive/negative categories). About half of those within every primary drug group also met criteria for alcohol abuse or dependence. Notably, as with abuse or dependence on the primary drug, there was no difference in rates of abuse or dependence diagnosis for secondary drugs among those classified as positive versus negative at baseline.

Table 3 also shows the percent qualifying for active use of a secondary drug at baseline, based on positive urine test, or, in the case of alcohol, self-reported use within the past 7 days. The majority of participants within each primary drug use category did not have evidence of active secondary drug use at the time of the baseline assessment. However, with the exception of primary opioid users, rates of active secondary drug use were higher among those positive for their primary drug than among those testing negative, with this difference being statistically significant for alcohol and stimulant users.

### Physical health, mental health, social adjustment, and 12-step participation

As shown in Table 4, physical health status (higher scores representing better health) was rated as worse among those positive for marijuana (positive N = 69; negative N = 81) and cocaine/stimulants (positive N = 65; negative N = 77). Among cocaine/stimulant users, those testing positive had lower social adjustment scores (*p* < .01) and higher rates of depression (positive = 46%; negative = 16%) and panic disorder (positive = 27%; negative = 11%) than those testing negative. Table 4 also shows a striking behavioral difference among the drug-positive versus -negative samples in their utilization of 12-step programs in the 90 days prior to baseline assessment. Twelve-step attendance was endorsed by 69% who were abstinent from alcohol at baseline, compared to 40% of those reporting alcohol use in the past 7 days. Mean days of attendance for alcohol users were 24.3 and 6.7, respectively (*p* <.05; not shown). Among cocaine/stimulant users, 74% of those testing negative at baseline reported 12-step attendance (compared to 49% of those testing positive). Mean days of attendance for cocaine/stimulant users were 19.1 and 13.2, respectively (difference not significant; not shown). There were no significant differences between positive versus negative primary marijuana or opioid users in percentage attending or mean days of attendance.

**Table 4:** Physical health, mental health, and social adjustment variables

	**Alcohol**			**Marijuana**			**Cocaine/Stimulants**			**Opioids**		
	**Neg N = 64**	**Pos N = 40**	**( *t, p *)**	**Neg N = 37**	**Pos N = 75**	**( *t, p *)**	**Neg N = 129**	**Pos N = 41**	**( *t, p *)**	**Neg N = 91**	**Pos N = 17**	**( *t, p *)**
Physical Health^a^	74.6	67.1	(1.75, 0.08)	81.4	69.1	(3.21, p < .01)	76.6	64.7	(3.45, <0.001)	73.0	67.1	(1.20, 0.23)
Social Adjustment^b^	2.2	2.3	(1.51, 0.13)	2.0	2.1	(0.51, 0.61)	2.2	2.5	(3.37, <0.01)	2.1	2.3	(1.57, 0.14)
			(χ2 [df], *p*)			(χ2 [df], *p*)			(χ2 [df], *p*)			(χ2 [df], *p*)
Depression^c^	21.9	30.0	(0.87 [1], 0.35)	10.8	17.3	(0.82 [1], 0.37)	16.3	46.3	(15.6 [1], <.001)	18.7	23.5	(0.21 [1], 0.64)
Anxiety^c^	29.7	20.0	(1.20 [1], 0.27)	29.7	28.0	(0.04 [1], 0.85)	23.3	31.7	(1.18 [1], 0.28)	31.9	35.3	(0.08 [1], 0.78)
Panic^c^	25.0	30.0	(0.31 [1], 0.58)	8.1	17.3	(1.72 [1], 0.19)	10.9	26.8	(6.33 [1], 0.02)	16.5	35.3	(3.24 [1], 0.07)
12-Step^d^	68.8	40.0	(8.34 [1], <0.01)	29.7	17.3	(2.26 [1], 0.13)	73.6	48.8	(8.79 [1], <0.01)	76.9	64.7	(1.14 [1], 0.29)

^a^Scores range from 0 to 100, with higher scores representing better health; means scores are shown.

^b^Mean item score across 54 items; five-point item scales range from one (indicating higher functioning) to five (indicating lower functioning).

^c^Percent with probable diagnosis from Patient Health Questionnaire (PHQ).

^d^Proportion of patients reporting attendance.

## Discussion

This study provides new data on characteristics and behaviors of those who are seeking treatment for substance use and are assessed early in their treatment episode. Data were derived from a large NIDA CTN study conducted at 10 regionally diverse community treatment programs and thus, may have a level of generalizability for similar substance abuse treatment-seeking populations across the US. Specifically, these results provide insight into the current drug use profiles of patients entering programs and seeking treatment for alcohol, cocaine/stimulants, marijuana, and opioids. These findings also stand to help treatment providers better understand the multiple health needs and challenges, as well as resilience behaviors, among patients who are initiating substance abuse treatment.

### Early treatment abstinence and drug use

The results of this study confirm and extend previous observations that a high proportion of stimulant abusers test negative for their primary drug of abuse at treatment entry or shortly thereafter [1,2,6,13,29,30]. Our analysis of data from this large patient sample also suggests that early treatment abstinence is fairly common among substance users seeking treatment in community outpatient counseling treatment programs, irrespective of the primary endorsed drug of abuse, including those who identify primary problems with opioids, cocaine/stimulants, marijuana, and alcohol. This study also demonstrates the discrepant data that emerge when drug use status is based on different assessment methods and timeframes. Urine testing at treatment entry provides insight only into very recent use, versus abstinence, and does not capture use within the past 30 days, a commonly assessed timeframe and one that yields higher prevalence of recent drug use (Table 2). It is clear from previous research in the field that, for stimulant users, the urine test result provides important prognostic information [1,2,6,13,29,30]; whether this is true for other drugs of abuse remains to be seen. Also of interest for future research is whether self-report adds any prognostic value to the urine test results, and if so, what timeframe might be important. One recommendation for future research and clinical practice is to obtain more detailed information about patterns of pre-treatment drug use [17,18], which can be obtained using validated TLFB methods [24].

This study also demonstrates important similarities across primary substances of abuse in characteristics of those testing positive versus negative for their primary drug early in treatment. In particular, there was no distinction across these groups in the DSM classification of past year abuse or dependence (Table 3). This suggests that the DSM criteria may not be useful for differentiating patients at intake with regard to their characteristics or behaviors. However, assessment of current rather than past year dependence may provide data more congruent with behavior at the start of treatment.

### Co-occurring problems

The study adds to the existing literature by identifying a profile of adverse physical, mental health, and behavioral characteristics associated with active versus inactive early treatment substance use. Specifically, we found poorer physical health status (marijuana and cocaine/stimulant users) and poorer mental health and social adjustment scores, with evidence of elevated rates of depression and panic disorder, among cocaine/stimulant users testing positive for their primary drug early in treatment (Table 4). Thus, evidence of active drug use early in treatment indicates a higher likelihood of, and may be a marker for, concurrent health/mental health, and/or social adjustment problems. This need is relevant to current thinking about the integration of primary and mental health care within drug and alcohol treatment settings (or vice versa) [19], a model from which patients with active substance use and co-morbid physical and/or mental health conditions could greatly benefit. Given the higher likelihood of co-occurring health and mental health problems for those with active use, it will be important for providers to attend to drug use behavior at, and shortly after, treatment entry and to broaden assessments for those testing positive to include additional health and mental health domains in anticipation of possible health and mental health care needs of these patients.

### Early treatment help-seeking

This study provides new data about the resilience of those who test drug-negative early in treatment by showing that many participants are already attending 12-step programming prior to treatment entry, especially for primary alcohol and stimulant users. In specific terms, a substantial percentage of alcohol (69%) and stimulant (74%) abusers who had stopped using their primary drug by the early treatment assessment reported recent involvement in 12-step programs. Mean days of 12-step program attendance in the past 90 days among negative stimulant and alcohol users suggests that self-help treatment may have been underway for some time before the formal treatment program had begun. Such proactive behavior represents an important strength that these individuals bring to the current treatment episode [31-33].

### Limitations

The main limitation of the study is that individuals included in the analysis all had agreed to participate in a research study and were limited to those (including those endorsing alcohol as their primary drug) who reported some drug use within the past 30 days. The sample thus may not be fully representative of all individuals who abuse substances and seek treatment. However, given the *WEB-TX* all-comers sample was recruited from regionally diverse settings across the US, this study may better represent seekers of treatment than other samples that have recruited more narrowly focused populations. This study was also limited in that the baseline assessment was conducted after treatment entry and thus cannot differentiate between pre-treatment and early treatment behavior change. Future research that examines baseline-use status should make efforts to distinguish between pre- and early treatment behaviors. Finally, while this paper has primarily focused on creating a greater understanding in the field of the health and behavior characteristics of patients with and without active use of their primary drug at treatment entry, an important aspect of these data is their possible prognostic capability. Future research should examine the influence of baseline abstinence on substance use outcomes within each of the primary drug use groups, as well as the prognostic contribution of other health and behavior variables associated with active drug use at entry. Results of such analyses will be a useful addition to those presented herein.

## Conclusions

Our study demonstrates that early treatment abstinence is common among substance users entering treatment with a range of primary substances of abuse including alcohol, marijuana, cocaine/stimulants, and opioids. The study further demonstrates that those who are drug-negative early in treatment are more likely to be attending 12-step programming, while those positive at treatment entry are more likely to have secondary drug use, as well as co-morbid physical and mental health problems. These findings provide important information for clinicians to better understand the health issues and substance use profiles among patients seeking treatment and to take a proactive approach to interdisciplinary treatment needs of patients.

## Competing interests

The authors have no competing interests to declare.

## Authors’ contributions

GC and MS conceptualized, interpreted analyses, and wrote large portions of the manuscript. EN was the Principal Investigator on the parent clinical trial and helped to conceptualize the current project and provided critical feedback on the manuscript. MCH conducted the statistical analyses and aided in the interpretation of analyses. AC was the project manager on the parent clinical trial and helped to conceptualize the current project and aided in the interpretation of analyses and provided critical feedback on the manuscript. Each author has approved of the final version of this manuscript.

## References

[B1] Alterman A Kampman KM Boardman C Cacciola J Rutherford M McKay J Maany I A cocaine-positive baseline urine predicts outpatient treatment attrition and failure to attain initial abstinence Drug Alcohol Depend 1997 46 79 85 10.1016/S0376-8716(97)00049-5 9246555

[B2] Bisaga A Aharonovich E Garawi F Levin FR Rubin E Raby WN Vosburg SK Nunes EV Utility of lead-in period in cocaine dependence pharmacotherapy trials Drug Alcohol Depend 2005 77 7 11 10.1016/j.drugalcdep.2004.06.007 15607836

[B3] Borders T Booth B Falck R Leukefeld C Wang J Carlson R Longitudinal changes in drug use severity and physical health-related quality of life among untreated stimulant users Addict Behav 2009 34 959 964 10.1016/j.addbeh.2009.06.002 19560873 PMC2726274

[B4] Ehrman RN Robbins SJ Cornish JW Results of a baseline urine test predict levels of cocaine use during treatment Drug Alcohol Depend 2001 62 1 7 10.1016/S0376-8716(00)00137-X 11173162

[B5] Falck R Wang J Carlson R Health status of illicit stimulant drug users in rural Ohio J Psychoactive Drugs 2007 4 401 405 18284105 10.1080/02791072.2007.10399901

[B6] Kampman K Volpicelli J Mulvaney F Rukstalis M Alterman A Pettinati H Weinrieb R O’Brien C Cocaine withdrawal severity and urine toxicology results from treatment entry predict outcome in medication trials for cocaine dependence Addict Behav 2002 27 251 260 10.1016/S0306-4603(01)00171-X 11817766

[B7] Peirce JM Petry NM Roll JM Kolodner K Krasnansky J Stabile PQ Brown C Stitzer ML Correlates of stimulant treatment outcome across treatment modalities Am J Drug Alcohol Abuse 2009 35 48 53 10.1080/00952990802455444 19152207 PMC2722066

[B8] Penberthy J Ait-Daoud N Vaughan M Fanning T Review of treatment for cocaine dependence Curr Drug Abuse Rev 2010 3 49 62 10.2174/1874473711003010049 20088819

[B9] Petry NM Barry D Alessi SM Rounsaville BJ Carroll KM A randomized trial adapting contingency management targets based on initial abstinence status of cocaine-dependent patients J Consult Clin Psychol 2012 80 276 285 22229758 10.1037/a0026883 PMC3668312

[B10] Plebani JG Kampman KM Lynch KG Early abstinence in cocaine pharmacotherapy trials predicts successful treatment outcomes J Subst Abuse Treat 2009 37 313 317 10.1016/j.jsat.2009.02.001 19339141 PMC2762233

[B11] Schierenberg A van Amsterdam J van den Brink W Goudriaan AE Efficacy of contingency management for cocaine dependence treatment: a review of the evidence Curr Drug Abuse Rev 2012 5 320 331 23244344 10.2174/1874473711205040006

[B12] Sofuoglu M Gonzalez G Poling J Kosten TR Prediction of treatment outcome by baseline urine cocaine results and self‒reported cocaine use for cocaine and opioid dependence Am J Drug Alcohol Abuse 2003 29 713 727 10.1081/ADA-120026256 14713135

[B13] Stitzer ML Petry N Peirce J Kirby K Killeen T Roll J Hamilton J Stabile PQ Sterling R Brown C Kolodner K Li R Effectiveness of abstinence-based incentives: interaction with intake stimulant test results J Consult Clin Psychol 2007 75 805 811 17907862 10.1037/0022-006X.75.5.805

[B14] Sullivan LE Botsko M Cunningham CO O’Connor PG Hersh D Mitty J Lum PJ Schottenfeld RS Fiellin DA BHIVES Collaborative The impact of cocaine use on outcomes in HIV-infected patients receiving buprenorphine/naloxone J Acquir Immune Defic Syndr 2011 56 S54 S61 21317595 10.1097/QAI.0b013e3182097576 PMC3065971

[B15] Brensilver M Heinzerling KG Swanson AN Shoptaw SJ A retrospective analysis of two randomized trials of bupropion for methamphetamine dependence: suggested guidelines for treatment discontinuation/augmentation Drug Alcohol Depend 2012 125 169 172 10.1016/j.drugalcdep.2012.03.027 22534658 PMC3418457

[B16] Brensilver M Heinzerling KG Swanson AN Shoptaw SJ Placebo-group responders in methamphetamine pharmacotherapy trials: the role of immediate establishment of abstinence Exp Clin Psychopharmacol 2012 20 430 435 22867036 10.1037/a0029210 PMC4035480

[B17] Gueorguieva R Wu R Donovan D Rounsaville BJ Couper D Krystal JH O’Malley SS Baseline trajectories of heavy drinking and their effects on postrandomization drinking in the COMBINE study: empirically derived predictors of drinking outcomes during treatment Alcohol 2012 46 121 131 10.1016/j.alcohol.2011.08.008 21925828 PMC3266454

[B18] Gueorguieva R Wu R Donovan D Rounsaville BJ Couper D Krystal JH O’Malley SS Baseline trajectories of drinking moderate acamprosate and naltrexone effects in the COMBINE study Alcohol 2011 35 523 531 10.1111/j.1530-0277.2010.01369.x PMC3062945 21143249

[B19] Buck JA The looming expansion and transformation of public substance abuse treatment under the Affordable Care Act Health Aff 2011 30 1402 1410 10.1377/hlthaff.2011.0480 21821557

[B20] Pating DR Miller MM Goplerud E Martin J Ziedonis DM New systems of care for substance use disorders: treatment, finance, and technology under health care reform Psychiatr Clin of North Am 2012 35 327 356 10.1016/j.psc.2012.03.004 22640759

[B21] Campbell AN Nunes EV Miele GM Matthews A Polsky D Ghitza UE Turrigiano E Bailey GL VanVeldhuisen P Chapdelaine R Froias A Stitzer ML Carroll KM Winhusen T Clingerman S Perez L McClure E Goldman B Crowell AR Design and methodological considerations of an effectiveness trial of a computer-assisted intervention: an example from the NIDA Clinical Trials Network Contemp Clin Trials 2012 33 386 395 10.1016/j.cct.2011.11.001 22085803 PMC3268951

[B22] Campbell AN Nunes EV McClure EA Hu MC Turrigiano E Goldman B Stabile PQ Characteristics of an outpatient treatment sample by primary substance of abuse J Addict Med 2013 7 363 371 10.1097/ADM.0b013e31829e3971 24089040 PMC3790139

[B23] Hudziak J Helzer JE Wetzel MW Kessel KB McGee B Janca A Przybeck T The use of the DSM-III-R Checklist for initial diagnostic assessments Compr Psychiatry 1993 34 375 383 10.1016/0010-440X(93)90061-8 8131381

[B24] Sobell MB Sobell LC Bogardis J Leo G Skinner W Problem drinkers’ perceptions of whether treatment goals should be self-selected or therapist-selected Behav Ther 1992 23 43 52 10.1016/S0005-7894(05)80307-7

[B25] The EuroQol Group EuroQol – a new facility for the measurement of health-related quality of life Health Policy 1990 16 199 208 10109801 10.1016/0168-8510(90)90421-9

[B26] Spitzer RL Kroenke K Williams JB Validation and utility of a self-report version of PRIME-MD: the PHQ primary care study JAMA 1999 282 1737 1744 10.1001/jama.282.18.1737 10568646

[B27] Weissman M Social Adjustment Scale – Self Report 1990 North Tonawanda, NY: MHS

[B28] SAS SAS 9.2 2009 Cary, NC, USA: SAS Institute Inc

[B29] Bisaga A Aharonovich E Garawi F Levin FR Rubin E Raby WN Nunes EV A randomized placebo-controlled trial of gabapentin for cocaine dependence Drug Alcohol Depend 2006 81 267 274 10.1016/j.drugalcdep.2005.07.009 16169160

[B30] Ghitza UE Epstein DH Preston KL Self-report of illicit benzodiazepine use on the Addiction Severity Index predicts treatment outcome Drug Alcohol Depend 2008 97 150 157 10.1016/j.drugalcdep.2008.04.003 18499354 PMC2553754

[B31] Fiorentine R Counseling frequency and the effectiveness of outpatient drug treatment: revisiting the conclusion that “more is better” Am J Drug Alcohol Abuse 2001 27 617 631 10.1081/ADA-100107659 11727880

[B32] Fiorentine R Hillhouse MP Drug treatment and 12-step program participation: the additive effects of integrated recovery activities J Substance Abuse Treat 2000 18 65 74 10.1016/S0740-5472(99)00020-3 10636609

[B33] Fiorentine R Hillhouse MP Why extensive participation in treatment and twelve-step programs is associated with the cessation of addictive behaviors: an application of the addicted-self model of recovery J Addict Dis 2003 22 35 55 10.1300/J069v22n01_03 12661978

